# Immunization with Embryonic Stem Cells/Induced Pluripotent Stem Cells Induces Effective Immunity against Ovarian Tumor-Initiating Cells in Mice

**DOI:** 10.1155/2023/8188324

**Published:** 2023-11-03

**Authors:** Fengsheng Yu, Zujuan Zhang, Xiaohong Chang, Xue Ye, Hongyan Cheng, Yi Li, Heng Cui

**Affiliations:** ^1^Center of Gynecologic Oncology, Peking University People's Hospital, Beijing 100044, China; ^2^Department of Obstetrics and Gynecology, The Affiliated Hospital of Qingdao University, Qingdao 266000, China; ^3^Department of Gynecology, Women's Hospital, School of Medicine, Zhejiang University, Hangzhou 310000, China; ^4^Department of Obstetrics and Gynecology, Peking University People's Hospital, Beijing 100044, China

## Abstract

Cancer stem cells (CSCs) express pluripotent markers and share many features with normal pluripotent stem cells. It is possible that immunity induced by embryonic stem cells (ESCs) and induced pluripotent stem cells- (IPSCs-) based vaccines may selectively target CSCs. In our study, cells expressing the pluripotent marker CD133 in the murine ovarian cancer cell-line ID8 were isolated and identified as CSCs. We investigated the preventive efficacy of ESCs and IPSCs-based vaccines against the development of ovarian cancer *in vivo* and evaluated the humoral and cellular immunities targeting CSCs *in vitro*. Our study showed that preimmunization with both mouse-derived embryonic stem cells (mESCs) and mouse-induced pluripotent stem cells (mIPSCs) lysates, combined with an immunostimulatory adjuvant CpG, elicited strong humoral and cellular responses. These responses effectively suppressed the development of CSC-derived tumors. Immune sera collected from mESCs and mIPSCs-vaccinated mice contained antibodies that were capable of selectively targeting CSCs, resulting in the lysis of CSCs in the presence of complement. Cytotoxic *T*-lymphocytes generated from splenocytes of mESCs and mIPSCs-vaccinated hosts could secrete interferon- (IFN-) *γ* in response to CSCs and kill CSCs *in vitro*. These findings indicate that vaccines based on mESCs and mIPSCs can elicit effective antitumor immunities. These immunities are related to the conferring of humoral and cellular responses that directly target CSCs.

## 1. Introduction

Due to its late detection and high rate of recurrence after first-line treatment, ovarian cancer is the most lethal gynecological malignancy [1]. The gold-standard treatment for ovarian cancer patients consists of debulking surgery and a combination of taxane and platinum-based chemotherapy. After initial treatment, the majority of patients eventually experience relapse and develop chemoresistant tumors. The presence of small populations of cancer stem cells (CSCs) is now proposed to be the cause of tumor recurrence and reduced therapeutic efficacy. Due to the epigenetic plasticity and inherent resistance mechanisms of CSCs, conventional therapies are often insufficient to eliminate these populations. Therefore, there is an urgent need for effective targeted treatment to eliminate CSCs [2]. Recent studies have shown that immune-based methods may be up-and-coming for targeting CSCs. One strategy is to target CSCs with the monoclonal antibodies, while the other is to induce effective immune responses against these cells [3].

Schöne [4] demonstrated over a century ago that animals inoculated with embryonic/fetal tissue were able to reject transplanted tumors, which laid the foundation for using embryonic material as an antitumor vaccine in animal experiments [5]. Subsequent studies revealed that immunizing animals with embryonic materials can trigger protective humoral and cellular immune responses against transplantable tumors and carcinogen-induced tumors. This supports the notion that antitumor immunity may be induced by the antigens shared between embryonic material and cancer cells [6–13]. The majority of CSC-identified markers are derived from surface markers found on human embryonic stem cells (hESC) or adult stem cells. About 73% of the current CSCs' surface markers appear to be present on embryonic stem cells (ESCs) or adult stem cells, while normal tissue cells rarely express them [14].

In this study, we utilized mESCs and mIPSCs as immunogenic agents. The vaccines, inoculated cancer cells, and tumor-bearing animals are congeneric, which might have avoided the interference of heterologous immune responses. We initially isolated and identified a subpopulation expressing CD133 in the ID8 cell line. This subpopulation fulfills the criteria for CSCs and possesses the ability to initiate tumors both *in vitro* and *in vivo*. The immune responses against ovarian CSCs induced by vaccines based on mESCs and mIPSCs were then evaluated in mice. Our study showed that vaccines based on mESCs and mIPSCs can effectively induce protective antitumor immunity. This immunity is associated with the activation of both humoral and cellular responses that specifically target CSCs.

## 2. Materials and Methods

### 2.1. Animals

Pregnant ICR mice (Vital River Laboratories, Beijing, China) at 13.5-day post-coitum were kept under specific pathogen-free (SPF) conditions for preparing mouse embryonic fibroblasts (MEFs). Nonobese diabetes server combined immune deficiency mice (NOD/SCID) and 6-week-old female C57BL/6 mice (Vital River Laboratories, Beijing, China) were reared and maintained under SPF conditions at the Animal Laboratory of Peking University People's Hospital (Beijing, China).

### 2.2. Cell Lines and Cell Culture

The murine ovarian cancer cell-line ID8, which was generously provided by Professor Katherine Roby (Center for Reproductive Sciences, University of Kansas Medical Center, USA), is a mouse ovarian surface epithelial cancer cell line derived from ovarian surface epithelial cells of C57BL/6 mice [15]. Cells were cultured in Dulbecco's Modified Eagle's Medium (DMEM)/high glucose, supplemented with 5% fetal bovine serum (FBS) and 1% insulin–transferrin–selenium (ITS, Sigma). For sphere formation, ID8 cells were cultured in the serum-free DMEM/F12 medium, supplemented with 20 ng/ml mouse recombinant epidermal growth factor (mEGF, Life Technologies), 2% B27 supplement without vitamin A (Life Technologies), 10 ng/ml mouse recombinant basic fibroblast growth factor (mbFGF, Life Technologies), 1% ITS, 100 IU/ml penicillin, and 100 *μ*g/ml streptomycin. The ultralow-attachment 6-well plates (Corning) were used to reduce cell adherence and support growth as spheres. The mouse-embryonic stem cell (mESC) line IVP-ES1 was derived from fertilized embryos of B6D2 (C57BL/6 × DBA) F1 females [16]. The mouse-induced pluripotent stem cell (mIPSC) line IP14D-1 was derived from B6D2 F1 (F1 of C57BL/6J 3 DBA/2J) mouse embryonic fibroblasts [17]. These cell lines were generously provided by Professor Qi Zhou (Institute of Zoology of Chinese Academy of Sciences). Cells were cultured in DMEM/high glucose (4.5 g/L), supplemented with 20% FBS, 1,000 IU/ml leukemia inhibitory factor (LIF, Life Technologies), 0.1 mM *β*-mercaptoethanol (Sigma), 2 mM L-glutamine, 1% nonessential amino acids (NEAA, Life Technologies), 50 IU/ml penicillin, and 50 *μ*g/ml streptomycin, in 0.1% gelatin-coated plates at 37°C with feeder layer cells in humidified air with 5% CO_2_. MEFs were employed as feeder layer cells and were cultured in DMEM/high glucose, supplemented with 10% FBS, and inactivated with 10 *μ*g/ml mitomycin C (Sigma) for 3 hr before use. To separate mESCs/mIPSCs from feeders when they were used to prepare cell lysates, the cell suspension was transferred into a 10-cm tissue culture dish (without gelatin-coating) filled with 10 ml fresh ESC-culture medium and incubated for 40 min. MEFs should begin to attach 15 min after plating.

### 2.3. Sphere Formation Assay

**Figure 1:**
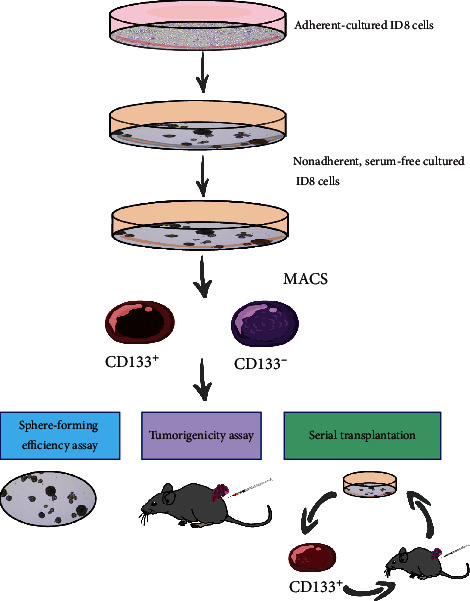
Isolation and characterization of CSCs from the ID8 cell line.

For sphere formation, ID8 cells cultured under adherent conditions were collected and washed to remove serum, then suspended in a serum-free medium. These cells were subsequently cultured at a density of 2 × 10^4^ per well in ultralow-attachment 6-well plates. A new medium was added every 2–3 days as soon as the supernatants in each well were gently aspirated out. When the spheres reached a diameter of approximately 50 *μ*m, they were collected by gentle centrifugation. Subsequently, they were dissociated with trypsin-EDTA and mechanically disrupted using a pipette. The single cells were then centrifuged to remove the enzyme and recultured in serum-free medium to reform spheres. The spheres would be passaged every 5–7 days once they reached a diameter of 50 *μ*m (Figure 1).

### 2.4. Magnetic-Activated Cell Sorting (MACS)

For magnetic separation, cells were sorted immediately after enzymatic dissociation using the Dead Cell Removal Microbead Kit (Miltenyi Biotec) according to the manufacturer's instructions. Viable cells were initially labeled using anti-CD133 IgG (anti-Mouse CD133, Rat IgG1, eBioscience). Then, anti-rat IgG MicroBeads (Miltenyi Biotec) were used at a concentration of 2 *μ*l per 1 × 10^6^ cells to isolate CD133-positive and CD133-negative populations. This isolation was achieved through double passage using the MidiMACS system (Miltenyi-Biotec). The cell suspension was applied to the LS column and then loaded into the MACS separator station. The magnetic sorting buffer was used to elute the CD133-negative cells from the LS column, and the MACS separator was used to elute the CD133-positive cells. The procedure was carried out in a sterile manner to ensure that the cells could be used for the future investigations.

### 2.5. Sphere-Forming Efficiency Assay

MACS-sorted CD133-positive and CD133-negative ID8 cells were dissociated into single-cell suspensions, and 500 cells per well were plated in ultralow attachment 24-well plates. The cells were cultured at 37°C in 5% CO_2_ for 12 days, and the serum-free medium was replaced twice a week. Spheres larger than 50 *μ*m in diameter were counted in each well using inverted phase-contrast microscopy. All experiments were conducted in triplicate.

### 2.6. Tumorigenicity Assay

Twenty NOD/SCID female mice were randomly divided into four groups. Trypan blue staining was performed to measure cell viability, and various numbers (100, 500, 2,000, and 10,000) of viable CD133-positive and CD133-negative cells were subcutaneously injected into the right and left flanks of NOD/SCID mice separately in phosphate buffer solution (PBS)/Matrigel (BD Biosciences, 1 : 1) using 100 *μ*l microsyringe. The tumor-bearing mice were observed twice a week. The endpoint was designated as any tumor that reached a diameter of 15 mm in any dimension. At that time, tumors were harvested from the euthanized mice for further investigation.

### 2.7. Serial Transplantation

Sorted CD133-positive ID8 cells were resuspended in PBS with Matrigel (1 : 1). Immediately after sorting, a 100 *µ*l solution containing 200 cells was injected subcutaneously into the flanks of 6-week-old female NOD/SCID mice. The mice were checked twice a week for the development of palpable tumors and were euthanized 18 weeks after being inoculated. The subcutaneous tumors were harvested and dissociated into a single-cell suspension. To begin, tumor tissues were mechanically dissociated into less than 1-mm fragments by gentle trituration, with all visible clumps removed, then digested at 37°C for 30 min with 1.6 mg/ml collagenase type I (Sigma) and 20 *µ*g/ml hyaluronidase (Sigma). To achieve dissociation into single cells, 0.2 g/ml trypsin was employed for 10 min on occasion. To eliminate any leftover aggregates, the cells were filtered through consecutive 75 *µ*m cell strainers. Filtered cells were suspended in PBS supplemented with 1% FBS. CD133-positive cells were sorted by MACS and reinoculated into NOD/SCID mice. Tumor formation was assessed later.

### 2.8. Flow Cytometry

CD133 expression was detected by flow cytometry in the primary ID8 cell line, spheroid-derived cells, and transplanted tumor-derived cells. The cells were labeled with rat anti-mouse CD133-PE antibody (Clone:315-2C11, Rat IgG2a, *λ*, BioLegend) or PE isotype control (rat IgG2a, *κ*, BioLegend).

### 2.9. Vaccine Preparation

**Figure 2:**
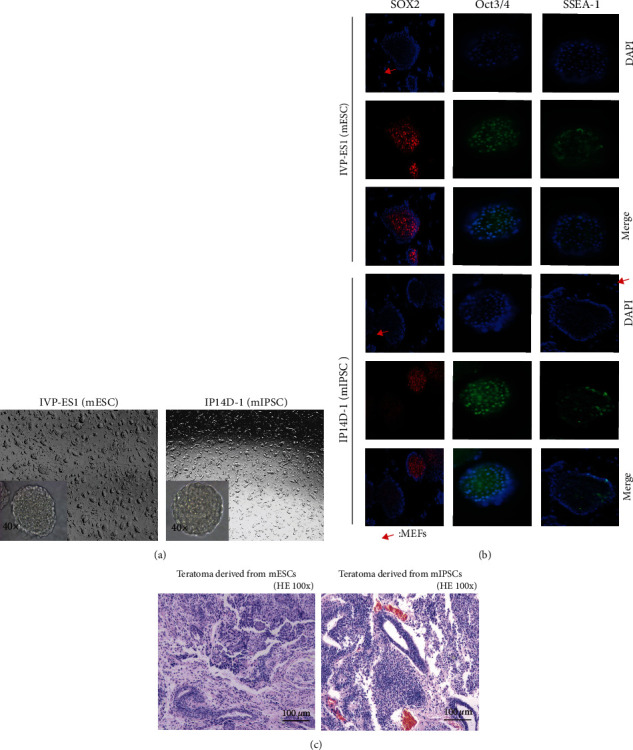
Culture and identification of mESCs and mIPSCs. (a) The culture was maintained with an ESC-conditioned medium, and images of typical mESCs and mIPSCs colonies were taken on Day 4 (magnification, ×40). Self-renewing mESCs/mIPSCs colonies have a spherical, packed appearance. (b) Pluripotency markers SOX2, Oct3/4, and SSEA-1 were stained in mESCs and mIPSCs colonies. Cell nuclei were visualized using the DNA-intercalating dye 40, 6-diamidino-2-phenylindole (DAPI). These pluripotency markers were positively expressed in mESCs and mIPSCs colonies but not in MEFs. (c) H&E (hematoxylin and eosin) staining revealed the presence of all three primary germ layer derivatives in intramuscular teratomas (bar, 100 *μ*m). Teratoma development was observed weekly after the mESCs/mIPSCs suspensions (100 *μ*l, 5 × 10^6^ cells with Matrigel™) were administered intramuscularly in NOD/SCID mice. Teratomas of 1 cm in diameter were collected and processed for histopathology.

mESCs and mIPSCs were cultured in an ESC-conditioned medium (Figure 2(a)). Pluripotency markers SOX2, Oct3/4, and SSEA-1 were expressed positively in colonies of mESCs and mIPSCs but not in MEFs (Figure 2(b)). ESCs and IPSCs are pluripotent cells with unlimited self-renewal ability, which can differentiate into cells representing three embryonic layers. The formation of teratoma in mice is used as an important *in vivo* method for detecting pluripotency, combined with the detection of pluripotency marker expression in vitro. This is the most rigorous method available for testing the pluripotency of isolated and cultured ESCs and IPSCs. To detect the pluripotency of IVP-ES1 and IP14D-1 cell lines, a teratoma formation assay was performed, and the expression of stem cell markers was analyzed (Figure 2(b) and 2(c)).

To generate the cell lysates, IVP-ES1, IP14D-1, and MEFs were collected and washed twice with PBS. Cells were then resuspended in PBS (5 × 10^7^ cells per ml) and lysed by 10 cycles of freezing at −80°C for 15 min, thawing at 37°C in a water bath for 15 min. Trypan blue exclusion confirmed complete cell death. The freeze–thaw cycles were repeated if any viable cells remained. The cell lysates were stored at −80°C for later use. Purified CpG ODN 1826 (5′-TCCATGACGTTCCTGACGTT-3′) was synthesized by Sangon Biotech (Shanghai, China) and was used as an adjuvant. CpG ODN 1826 was reconstituted in aseptic pyrogen free water at a concentration of 20 mg/ml and stored at −80°C for future use. Each vaccine dose (100 *µ*l of cell lysate or PBS supplemented with 20 *µ*g of CpG ODN 1826) was administered via subcutaneous injection above the shoulder using a 25-gauge needle.

### 2.10. Immunization Protocol and Tumor Challenge

**Figure 3:**
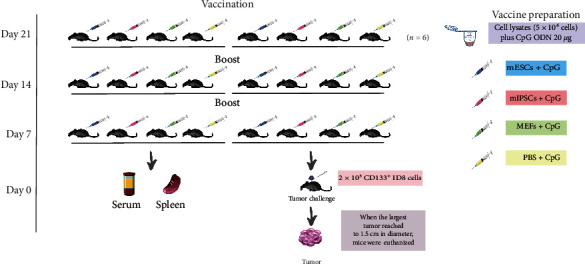
Immunization protocol, tumor challenge, and serum/splenocyte collection.

Twenty-four female 6-week-old C57BL/6 mice were randomly distributed into four groups (*n* = 6 mice per group) and were immunized with lysates of mESCs plus CpG ODN, lysates of mIPSCs plus CpG ODN, lysates of MEFs plus CpG ODN, and PBS plus CpG ODN separately. The mice were subcutaneously vaccinated on Days 21, 14, and 7. Another batch of 24 C57BL/6 mice was also grouped and immunized in the same way. All mice were anesthetized by intraperitoneal injection of ketamine-xylazine cocktail solution before immunization. On Day 0, one batch of mice was challenged with CD133-positive ID8 cells, and the other batch of mice was sacrificed to collect sera and splenocytes. Immediately after sorting, CD133-positive ID8 cells were resuspended in PBS at 4 × 10^6^ ml. About 2 × 10^5^ cells (50 *μ*l) mixed with 50 *μ*l of matrigel were injected subcutaneously into the right flank using a 100 *μ*l microsyringe. Tumor-bearing mice were monitored daily. Tumor growth was recorded weekly by measuring the diameter in two dimensions using a caliper. The formula for calculating tumor volume is (1/6)*π* × length × width^2^. The endpoint was defined as a tumor with a diameter of 15 mm in any dimension, and the animals were euthanized then (Figure 3).

### 2.11. Cytotoxic T-Lymphocyte Assay

The lactate dehydrogenase (LDH) release method (CytoTox96 Non-Radioactive Cytotoxicity Assay, Promega) was used for the cytotoxic T-lymphocyte (CTL) assay. Experiments were performed in accordance with the protocol of the manufacturer. The lytic activity of splenocytes against target cells (mitomycin-inactivated CD133-positive and CD133-negative ID8 cells) was measured after incubation for 4 hr at an effector to target cell ratio of 25 : 1, 50 : 1, and 100 : 1. The maximum release of LDH was achieved by culturing target cells with replenishment of lysis buffer. The target cells without splenocytes were used as negative controls. At the end of the incubation, 50 *μ*l aliquots from each well were transferred to a new 96-well plate. The substrate mix (50 *μ*l) was added to each well and then incubated for 30 min on a shaker in the dark. The reaction was stopped by adding 50 *μ*l of stop solution, and the absorbance at 490 nm was evaluated immediately. The cytotoxicity was calculated using the formula below (Equation (1)).

(1) $$\begin{array}{c} \text{\% Cytotoxicity}=\frac{\text{Experimental release}-\text{spontaneous release of effector cells}-\text{Spontaneous release of target cells}}{\text{Maximum LDH release}-\text{Spontaneous release }\left(\text{Negative control}\right)\text{ of target cells}}\times 100. \end{array}$$

### 2.12. Antibody-Mediated Complement-Dependent Cytotoxicity

Immediately after sorting, CD133-positive and CD133-negative cells were inoculated into a 96-well plate at a density of 5 × 10^3^ cells per well in DMEM supplemented with 1% FBS for 2 hr. Cells were gently washed with warm PBS, incubated with serum harvested from the immunized mice diluted 1 : 50 in PBS at 4°C for 1 hr, and then rewashed. This was followed by incubation with rabbit complement (Bio-Rad Laboratories, Inc.) diluted 1 : 25 in PBS at 37°C for 1 hr. Lysis was evaluated using the CytoTox96 Nonradioactive Cytotoxic Assay Kit. To achieve the maximum LDH release, the cells were treated with lysis buffer 45 min prior to centrifugation. The spontaneous release of LDH was obtained by cells without serum and complement. Plates were centrifuged at 250 g for 4 min and the supernatant of 50 *μ*l was transferred to the enzymatic assay plate and incubated at room temperature in the dark for 30 min with 50 *μ*l of substrate mix. A stop solution of 50 *μ*l was added to each well. Then the absorbance was measured at 490 nm using a plate reader (TECAN infinite M200N NanoQuant). In the new wells of each plate, an LDH positive control was added, and all tests were done in triplicate. The percentage of specific lysis was calculated according to the following formula (Equation (2)).

(2) $$\begin{array}{c} \text{\% Cytotoxicity}=\frac{\text{Experimental}-\text{Target spontaneous}}{\text{Target maximum}-\text{Target spontaneous}}\times 100. \end{array}$$

### 2.13. Interferon-Gamma Enzyme-Linked Immunosorbent Spot (ELISPOT) Assay

By using an IFN-*γ* ELISPOT kit (Mouse IFN gamma ELISPOT Ready-SET-Go!, eBioscience), we evaluated splenocytes collected from immunized mice to determine the presence of T cells capable of secreting IFN-*γ* in response to CD133-positive and CD133-negative cells. Nitrocellulose plates (Millipore, Milan, Italy) were coated overnight at 4°C with anti-IFN-*γ* capture monoclonal antibody (mIFN-*γ* kit; BD). Splenocytes were seeded at 2 × 10^5^ cells per well and stimulated with mitomycin-inactivated CD133-positive or CD133-negative cells (1 : 20) at 37°C for 48 hr, all conditions were performed in triplicate. Plates were then prepared using an AEC substrate according to the manufacturer's instructions, and the spots were quantified using a microplate reader. The number of spots was calculated by subtracting the number of spots in the medium (background) from the number of spots in the presence of stimuli.

### 2.14. Statistical Analysis

The results are reported as means and standard deviations for each data set. The significance of the differences in tumorigenicity, tumor size, cell lysis by antibodies or CTLs, and the number of spot-forming splenocytes was determined using either a two-sided Student's *t*-test or one-way analysis of variance (ANOVA) with Tukey's post hoc test. A value less than 0.05 (*P* < 0.05) was considered statistically significant. All analyses were performed using SPSS for Windows statistical software (version 20; SPSS Inc., USA).

## 3. Results

### 3.1. Isolation and Characterization of CSCs from the ID8 Cell-Line In Vitro

**Figure 4:**
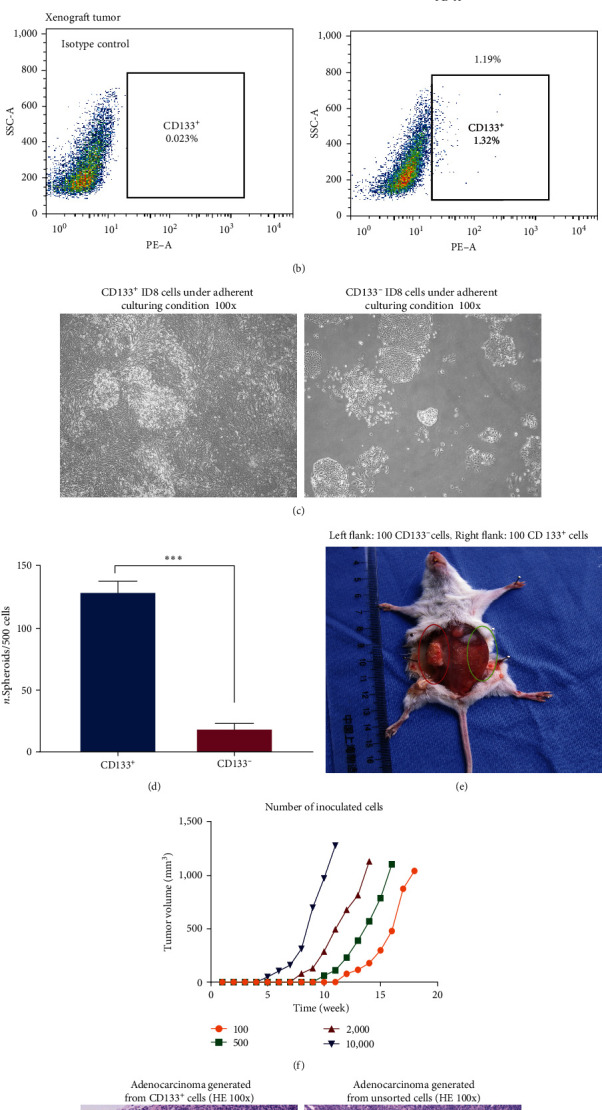
Self-renewal ability and tumorigenicity of CD133-positive ID8 cells. (a) ID8 cells formed floating tumor spheres about 50 *μ*m in diameter after 12 days of culture in serum-free medium. CD133-negative and CD133-positive ID8 cells were dissociated into single-cell suspensions, and 500 cells per well were incubated in ultralow attachment 24-well plates. (b) FACS analysis of CD133 expression in ID8 cell line, spheroid-derived cells, and transplanted tumor-derived cells. (c) 10^4^ CD133-positive and CD133-negative cells were inoculated in 6-cm collagen-coated dishes in DMEM medium containing 5% FBS for 7 days. The colonies derived from CD133-positive cells are fully fuzed, whereas the colonies derived from CD133-negative cells are isolated and scattered. (d) CD133-positive cells generated spheroids more efficiently and larger than CD133-negative cells ( ^*∗*^*P* < 0.05,  ^*∗*^ ^*∗*^*P* < 0.01, and  ^*∗*^ ^*∗*^ ^*∗*^*P* < 0.001, Student's *t*-test). (e) As few as 100 CD133-positive ID8 cells were sufficient to form tumors, while equal numbers of CD133-negative cells failed to generate tumors in NOD/SCID mice. (f) NOD/SCID mice were transplanted with varying numbers (100, 500, 2,000, and 10,000 cells) of CD133-positive ID8 cells. As few as 100 CD133-positive ID8 cells were able to consistently develop tumor xenografts. (g) H&E staining revealed that adenocarcinomas generated from CD133-positive cells in NOD/SCID mice show a high density of tumor cells and poor differentiation compared to tumors resulting from unsorted cells, which consisted mainly of nonproliferative hyalinized differentiation (bar, 50 *μ*m).

For enriching CSCs from the primary ID8 cell line, cells were plated in serum-free medium in ultralow attachment 6-well plates at a density of 20,000 cells per well. This density was chosen to ensure that the colonies could form separately from each other. Under such conditions, the cells grew in three dimensions without adhering to each other, forming structures known as spheres or spheroids (Figure 4(a)). Under the same culture conditions, single cells obtained from enzymatically dissociated spheroids formed secondary spheroids. This procedure was repeated for more than 20 passages, with cells being extensively amplified. CD133 expression in the ID8 cell line, spheroid-derived cells, and transplanted tumor-derived cells was analyzed using fluorescence-activated cell sorting (FACS) (Figure 4(b)).

One of the key features of CSCs is their self-renewal ability. CD133-positive and CD133-negative cells (10^4^ per dish) were inoculated into collagen-coated 6-cm dishes in DMEM medium containing 5% FBS for 7 days. The colonies derived from CD133-positive cells are fully fuzed, while the colonies derived from CD133-negative cells are isolated and scattered (Figure 4(c)). To functionally define the properties of stem cells, we conducted the sphere-forming assay using ID8 cells. The CD133-positive and CD133-negative ID8 cells sorted by MACS were inoculated into ultralow attachment 24-well plates at a density of 500 cells per well in a stem cell-conditioned culture (Figure 4(a)). Spheres larger than 50 *μ*m in diameter were counted in each well on Day 12. As a confirmatory experiment, we found that CD133-positive ID8 cells have a higher efficiency in sphere formation compared to their negative counterparts (Figure 4(d)).

### 3.2. CD133-Positive Cells Exhibit High-Tumorigenicity In Vivo

Accumulating evidence shows that CSCs have strong oncogenic potential. MACS-sorted CD133-positive cells and CD133-negative cells were subcutaneously injected into NOD/SCID mice in a limited dilution assay (100, 500, 2,000, 10,000 cells, *n* = 5) to test the hypothesis that CD133-positive cells are more tumorigenic due to their increased stem-like features. In the same animal, as few as 100 CD133-positive ID8 cells were sufficient for tumor formation (Figures 4(e) and 4(f), but at least 2 × 10^3^ CD133-negative ID8 cells were necessary (Table 1). H&E staining demonstrated that adenocarcinomas formed from CD133-positive cells had a higher tumor cell density and poorer differentiation than tumors derived from unsorted cells. The tumors derived from unsorted cells were primarily comprised of nonproliferative hyalinized fibroblasts (Figure 4(g)). These findings show that CD133-positive cells are more tumorigenic than CD133-negative cells. Serial transplantation of sorted cancer cells into immunodeficient animals is a functional test of the phenotype of CSCs. Three consecutive transplantations were performed to investigate the long-term tumor-forming capacity of CD133-positive cells in NOD/SCID animals. Our results showed that CD133-positive cells developed tumors with significant efficiency in each passage *in vivo*. These findings validate the presence of CSCs in the ID8 cell line and demonstrate that this subpopulation of cells can survive long-term passaging, including transplantation in NOD/SCID mice and *in vitro* cultures. These results imply that CD133 may be used as a reliable marker for enriching CSCs in the ID8 cell line.

### 3.3. mESCs/mIPSCs Vaccination Confers Significant Protective Antitumor Immunity

**Figure 5:**
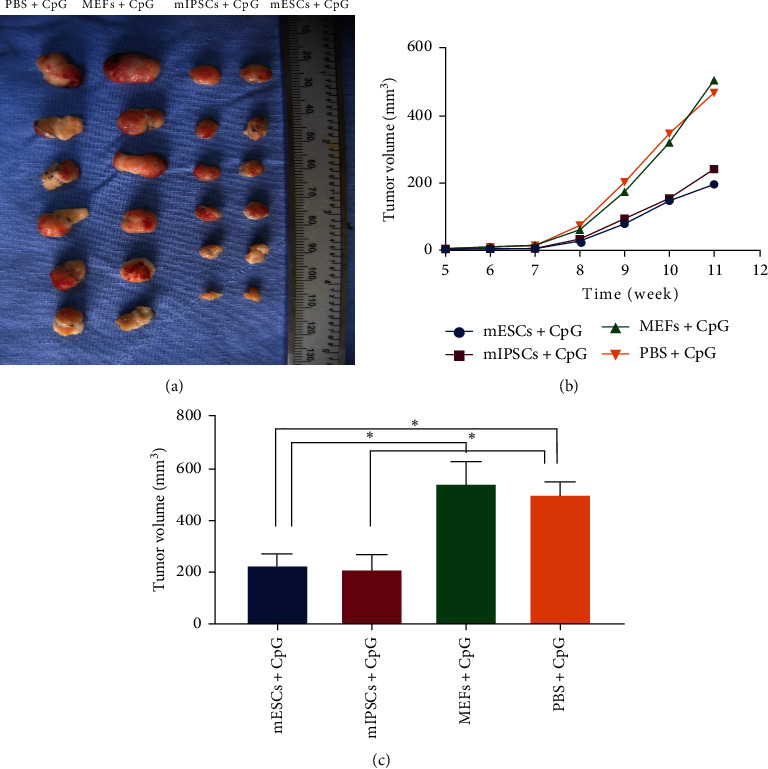
Vaccination with lysates of mESCs/mIPSCs induced significantly higher protective immunity against CSCs-derived tumors than controls *in vivo*. (a) Image of the tumors harvested from mice in all groups. (b, c) The tumor volume of mice immunized with mESCs/mIPSCs-based vaccine was significantly smaller compared with the control groups ( ^*∗*^indicates *p* < 0.05 compared to MEFs + CpG or PBS + cpG group, *n* = 6, error bars represent SD, one-way ANOVA).

In animal studies, ESCs and IPSCs have been shown to be effective vaccines in treating various cancers [8, 18], including the findings reported by our team [7, 9]. In this study, we evaluated the protective antitumor immunity induced by vaccination with cell lysates of mESCs and mIPSCs. We used lysates of MEFs or PBS as a negative control and employed CpG ODN as an adjuvant. We adopted mESCs and mIPSCs cell lysates, along with CpG ODN, to immunize mice three times, with a 7-day interval between adjacent immunizations. The control group received lysates of MEFs or PBS plus CpG ODN. Mice were challenged with CD133-positive ID8 cells 7 days after the final immunization. The immunization protocol and results are illustrated in Figures 3 and 5. The results indicate that vaccination with both mESCs and mIPSCs inhibits the development of tumors derived from CSCs. Although tumors did eventually develop in animals inoculated with mESCs/mIPSCs lysates, they were significantly smaller than those developed in the control groups (Figure 5(a)–5(c), *p* < 0.05).

### 3.4. mESCs/mIPSCs-Based Vaccine Induces Both an Antibody and a Cellular Response against CSCs

We collected splenocytes and sera from mice vaccinated with mESCs and mIPSCs to evaluate the specificity of the immune responses to CSCs and to investigate the potential mechanisms underlying the induction of protective antitumor immunity by mESCs and mIPSCs.

**Figure 6:**
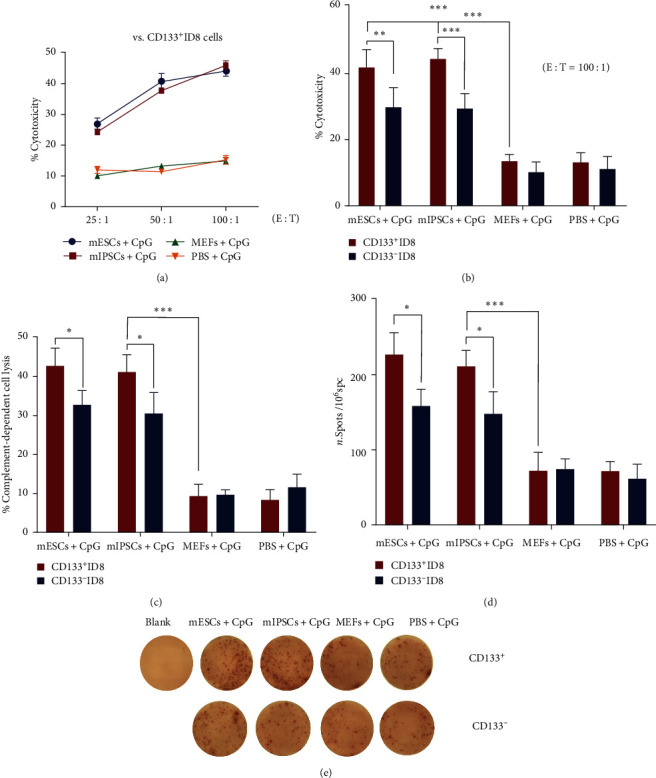
Vaccination with lysates of mESCs/mIPSCs generated lymphocytes that selectively target CSCs. (a, b) Targeting of CSCs and non-CSCs by mESCs/mIPSCs-primed CTLs. After 4 hr of incubation, the lytic activity of the splenocytes against target cells (CD133-positive and CD133-negative ID8 cells) was assessed using effector-to-target cell ratios of 25 : 1, 50 : 1, and 100 : 1. The killing of CSCs and non-CSCs mediated by CTLs was assessed using an LDH release assay. A higher percentage of cytotoxicity suggests a greater degree of cell lysis. (c) Complement-dependent cell lysis of CD133-positive cells was tested individually in serum from immunized mice. The CytoTox96 Nonradioactive Cytotoxicity Assay was used to assess the lysis of CD133-positive and CD133-negative ID8 cells. (d, e) Mouse splenocytes were assessed for the presence of T cells capable of secreting interferon- (IFN-) *γ* in response to CD133-positive and CD133-negative ID8 cells using an IFN-*γ* ELISPOT kit. ( ^*∗*^*P* < 0.05,  ^*∗*^ ^*∗*^*P* < 0.01, and  ^*∗*^ ^*∗*^ ^*∗*^*P* < 0.001, error bars represent standard deviation. Statistical analysis was performed using Student's *t*-test or one-way ANOVA).

The cytotoxic *T*-lymphocyte assay was employed in our investigation to provide evidence that the mESCs/mIPSCs-induced antitumor immunity is due to the direct targeting of CSCs. In this study, mESCs/mIPSCs primed CTLs efficiently destroyed CSCs in the ID8 cell line, with a rate of approximately 50%. This was significantly higher compared to CTLs primed with MEFs or PBS, which resulted in a destruction rate of less than 20% (Figures 6(a) and 6(b)). CSCs were more effectively destroyed by mESCs/mIPSCs-primed CTLs than non-CSCs (Figure 6(b)).

We then tested the immunological significance of mESCs/mIPSCs-primed antibody binding to CSCs by evaluating the antibody-mediated complement-dependent cytotoxicity of CSCs. Immune sera collected from mESCs/mIPSCs-vaccinated hosts were significantly more effective in lysing CD133-positive ID8 cells compared to sera from MEFs-vaccinated or PBS-treated hosts. CD133-positive ID8 cells were more effectively lysed than their counterparts (Figure 6(c)).

Splenocytes were harvested 7 days after the last vaccination and co-incubated with CD133-positive and CD133-negative cells to measure IFN-*γ* secretion using the ELISPOT assay. This was done to determine the presence of T cells capable of secreting IFN-*γ* in response to CD133-positive and CD133-negative cells. Splenocytes from mESCs/mIPSCs vaccinated hosts showed an increase in IFN-*γ* spots compared to the all control groups. Vaccination with mESCs/mIPSCs resulted in a higher number of T-cells specific to CSCs than T-cells that were not specific to CSCs (Figures 6(d) and 6(e)). These findings indicate that the secretion of IFN-*γ* in response to CSCs is specific, suggesting the induction of targeted immune responses to CSCs as a direct result of mESCs/mIPSCs-lysate vaccination.

## 4. Discussion

Despite the advancements in surgery and chemotherapy, the 5-year survival rate for all types of epithelial ovarian cancer is only 47% [19]. Therefore, ovarian cancer is still the deadliest gynecological cancer [1]. According to the accumulating data, certain tumor cells can survive chemotherapy by activating self-renewal mechanisms that result in tumor development and clinical recurrence. These cells, known as “tumor-starting cells” (TICs) or “cancer stem cells” (CSCs), have been shown to exhibit innate resistance to conventional chemotherapy and enhanced tumor-initiating capacity [2]. CSCs have unique characteristics similar to ESCs or IPSCs, including the ability to self-renew, reform tumor mass, and maintain homeostasis [20, 21]. Eliminating CSCs is thought to have the potential to overcome chemoresistance and minimize mortality in the ovarian cancer.

Ovarian cancer is considered as a potentially immunoreactive tumor because the presence of tumor-infiltrating lymphocytes correlates with the improved clinical outcomes [22–24]. Immune targeting of CSCs shows immense opportunities for the objective of overcoming cancer resistance and treating a larger number of ovarian cancer patients. Due to its potential to possibly eradicate the micrometastases that tend to linger after first-line treatment, vaccination is believed to offer advantages over traditional treatments. However, there is limited experimental data on the direct targeting of CSCs through vaccine-induced immunotherapy. Many immunotherapeutic approaches are used to target CSCs. These immunotherapies are usually classified as “active” or “passive”. Active immunotherapies boost the immune system to eliminate CSCs, employing strategies such as antitumor vaccines and checkpoint blockade medications. Passive immunotherapies include the transfer of immune cells, genetic modification of immune cells, cytokine-induced killer (CIK) cells, *γδ*T cells, and monoclonal antibodies (mAb). The most researched immunotherapeutic approaches involve using CSC-based, dendritic cells (DCs), DNA-vaccines, T-cell mediated, and antibody-based immunotherapies. While significant progress has been made, numerous concerns remain unsolved. For example, although checkpoint inhibitors have shown long-term responses in certain patients, boosting response rates with combination therapy raises the risk of autoimmune effects on the skin, gastrointestinal system, liver, and endocrine systems. Even though many patients respond to chimeric antigen receptor T-cell (CAR-T) therapy, there are still many cases of relapse due to antigen escape as well as a lack of T-cell persistence. CAR-T treatment has not yet been verified as effective in solid tumors [25]. A vaccine based on oncofetal peptides can only target a single antigen. Because the cells that make up a tumor are extremely heterogeneous and have a high mutation rate, this type of vaccination may not offer patients effective and long-term protection. Vaccinations or redirected T-cells should target multiple antigens, including CSC-specific antigens. DCs, as professional antigen-presenting cells (APCs), may load with various antigens including peptides, RNA, and whole tumor antigens, and present them to immune effector T cells. Many DC-based vaccines against CSCs have been successfully tested in animals. A combination of CSC-specific DC vaccines with standard cancer therapies as well as immune checkpoint inhibitors might be essential in future research and application of CSC-targeted cancer immunotherapy [26]. CSC-specific markers expressed by tumorigenic cells differ among patients. Identifying and isolating CSCs in clinical specimens remains a significant challenge, despite the availability of preclinically established surface markers for CSCs and other molecules specific to CSCs. On the other hand, DC vaccination needs an effective and sufficient quantity of DCs to accomplish the desired benefits. However, developing functional DCs in most patients is challenging due to chemotherapy or the tumor's immunosuppressive status [27].

Several studies have shown that both ESCs and IPSCs share known and likely even unknown tumor-specific antigens (TSAs) and tumor-associated antigens (TAAs) with common cancers, but not with healthy tissues [14, 28, 29], Therefore, they could potentially be used as reagents to stimulate the immune system to target cancer. Over a century ago, Schöne [4] recognized that immunization with embryonic materials could lead to the rejection of transplanted tumors in animals [5]. Later studies have shown that animals immunized with ESCs/IPSCs can generate antitumor immunity against a wide range of malignancies, such as colon cancer, lung cancer, and ovarian cancer [7–10, 18].

CSCs express markers of pluripotency and possess many molecular characteristics of normal pluripotent cells. Based on the similarities between CSCs and ESCs/IPSCs, vaccines based on ESCs/IPSCs may offer a representative panel of antigens similar to those of CSCs. These vaccines have the potential to elicit immune responses that specifically target CSCs. In this study, we evaluated the effect of ESC/IPSC-based vaccination on preventing tumor development by targeting CSCs in a syngeneic mouse model. A population enriched with CSCs was isolated from the murine ovarian cancer cell line ID8. This enrichment was based on the expression of the pluripotent marker CD133, which has been identified as a marker of both normal stem cells in several organs and tumorigenic populations in multiple malignancies [30, 31]. Baba et al. [31] first identified CD133 as a marker of ovarian cancer CSCs. Further studies indicated that CD133 expression could define a tumor-initiating cell population in primary human ovarian tumors [30, 32]. The expression of CD133 in ovarian cancer samples was associated with poor prognosis, including shorter overall and disease-free survival [33]. Using this marker, we enriched CSCs from the ID8 cell line to investigate the immune strategies of specifically targeting tumor-initiating cells in immunocompetent hosts. The isolated cells were able to grow as spheroids under serum-free conditions and to develop subcutaneous tumors following injection of as few as 100 cells in the presence of matrix gel. On the contrary, even as many as 500 CD133-negative cells were not able to generate tumors under these conditions. The CD133-positive population formed spheres more efficiently *in vitro* and developed tumors much larger and more rapidly than their negative counterparts *in vivo*. These findings suggest that the CD133-positive population isolated from the ID8 cell line exhibits the characteristics of CSCs. Recent studies have shown that cancer stemness indices are higher in recurrent and metastatic tumors than in initial tumors, lending credence to the idea that CSCs play critical roles in cancer recurrence and metastasis [34, 35]. Our data show that adenocarcinomas derived from CD133-positive cells have a high density of tumor cells and poor differentiation compared with tumors resulting from unsorted cells, which mainly consist of nonproliferative fibrinoid components, indicating that tumors derived from CD133-positive ID8 cells have features of recurrent tumors in ovarian cancer patients.

To enhance ESC/IPSC vaccine-induced immunity, CpG-oligodeoxynucleotide (ODN) was included as an immune-stimulating adjuvant in our study. CpG ODN, a toll-like receptor 9 (TLR9) agonist, can improve the function of specialized APCs and boost the induction of specific cellular and humoral immune responses to vaccine antigens. Ongoing clinical studies have shown that CpG ODN is safe and well-tolerated when used as an adjuvant in humans and can enhance vaccine-induced immune responses [36].

Vaccination with lysates of irradiated mESCs/mIPSCs, combined with the immunostimulatory adjuvant CpG ODN, was administered three times weekly, leading to induced antibodies that selectively bound to CSCs. Immune sera from mice vaccinated with mESCs and mIPSCs contained high levels of antibodies that selectively targeted CSCs. This led to the lysis of CSCs in the presence of complement. Vaccination with mESCs/mIPSCs lysates also induced T cells that could recognize CSCs *in vitro*. CTLs generated from splenocytes collected from mESCs/mIPSCs-vaccinated mice were able to secrete IFN-*γ* in response to CSCs and were capable of killing CSCs *in vitro*, indicating that the induced immune responses are specific to CSCs. The humoral and cellular immune responses induced by mESCs/mIPSCs may target both CSCs and non-CSCs, but they tend to target CSCs more selectively. The immunized mice rejected transplanted ovarian CSCs, which also suggests that the induced immune responses were specific to CSCs and functional.

Our study provides direct experimental evidence that mESCs/mIPSCs vaccine-induced antibodies and T cells can selectively target and destroy CSCs, and this immunological targeting of CSCs is related to enhancing the antitumor immunity conferred by ESCs/IPSCs vaccine *in vivo*. Preimmunization of both mESCs and mIPSCs elicited stronger humoral and cellular responses targeting CSCs, which suppressed the development of tumors in inoculated hosts. These findings give direct evidence that an ESCs/IPSCs vaccine can elicit a robust antitumor effect by immunologically targeting CSCs.

Because normal stem cells are also present in some adult organs, attempts to use ESCs as an anticancer vaccination in humans have been hindered by safety concerns. On the other hand, ESCs are commonly derived from an unrelated donor. They tend to express mismatched major histocompatibility complex (MHC) and/or minor histocompatibility antigens (mHags), which can elicit alloimmune responses when transplanted into the host. Previous studies by our colleagues showed that immunization with human pluripotent stem cells does not result in a significant autoimmune response. Complete blood count (CBC) assays showed no differences among rats immunized with hESCs and controls. Creatinine and serum liver enzyme levels were normal in control rats and rats immunized with hESCs [9]. More importantly, no abnormalities were observed in the weight, hair, joint swelling, and neuromuscular tension of the animals. There was no marrow suppression or damage to liver and kidney function. Immunized rats and mice were generally healthy without clinical evidence of autoimmune diseases [6]. As an index for systemic autoimmunity, a semi-quantitative assay for antinuclear antibodies (ANAs) in the sera of hESCs-immunized mice was performed. None of the post-immunized mice developed any clinical signs of autoimmune diseases, such as alopecia, skin rash, arthritis, or any obvious organ malfunction. Moreover, immunoblot analysis was performed using these sera against the lysates of the kidney, spleen, and liver, and no reactivity was observed [7]. A recent study showed that, by tissue analysis, mice at different time points post vaccination did not show any increases in immune cells within heart and kidney tissues compared to negative control groups, nor were elevated levels of antinuclear antigen (ANA) IgG seen in serum from a vaccinated mice [10]. Ouyang et al. [13] assessed animal autoimmunity by measuring body weight, organ histology, and antinuclear antibody levels. All of these parameters were normal, indicating that vaccinated animals were free of severe toxicity and autoimmune [13]. In this study, MEFs used as feeders were derived from mice, so we investigated if autoimmunity was elicited in those postimmunized mice due to the presence of a minor amount of MEFs in the cell lysates. No autoimmune response was found in the postimmunized mice used to collect serum and splenocytes. These mice had normal diet, activity, weight, skin, and hair as well as no joint swelling or neuromuscular tension. The iPSC-based vaccine could break the self-tolerance of the immune system to oncofetal antigens yet did not induce significant autoimmunity, which was possibly due to the higher abundance of these oncofetal antigens in tumors than in organs' resident stem cells [13].

Despite numerous similarities, a single-cell study has shown that IPSCs are more heterogeneous and pluripotent than ESCs [37], which indicates that IPSCs may be a better substitute to induce antitumor immune responses than ESCs because they not only can provide a broader set of neoantigens for specialized APSs to the hosts but also have no ethical concerns related to ESCs [38]. The vaccination of IPSCs might disrupt the immune system's self-tolerance to oncofetal antigens but did not result in severe autoimmunity. This could be due to the higher concentration of these oncofetal antigens in tumors compared to resident stem cells in organs [13]. Autologous IPSCs offer the benefits of being readily available and patient-specific, with fewer ethical problems than ESCs. They may be easily created by transfecting four Yamanaka factors (Oct4, Sox2, Klf4, and c-Myc) into somatic cells, and the 4-in-1 CoMiP reprograming technique allows for the creation of IPSC clones in 2 weeks [39]. Late, researchers developed a low-cost device with a simple design that can manufacture a large number of high-purity IPSCs for therapeutic usage in about 20 days [40]. In this case, the clinical development of the autologous IPSC-based cancer vaccine is guaranteed and achievable. These advantageous properties make the autologous IPSC vaccine a potential alternative for individualized adjuvant immunotherapy following conventional cancer treatment. Kooreman et al. [10] previously demonstrated that, when used as an adjuvant, IPSC vaccination can suppress the recurrence of melanoma at the resection site and reduce metastatic tumor burden. As a preventive application, an IPSC-based vaccine can be used to treat high-risk populations with Lynch syndrome or pathogenic germline mutations of the BRCA1/2 gene. Because these populations are more likely to develop ovarian cancer in their lifetime, they may be suitable candidates for preventive cancer vaccines. Individuals in these high-risk groups who receive early immunization with IPSC-based vaccines may prime their immune systems against various forms of tumor antigens, leading to the creation of memory cells capable of triggering tumor-specific immune responses when cancer cells are encountered [38].

## 5. Conclusions

Our study provides direct experimental evidence that vaccination with mESCs/mIPSCs can induce significant protective antitumor immunity. This immunity is associated with the conferring of humoral and cellular responses that specifically target CSCs. These findings support the development of a novel form of cancer immunotherapy based on the development of an IPSC vaccine capable of selectively targeting CSCs. This approach is particularly beneficial for patients who do not respond to immune checkpoint inhibitors or neoantigen vaccines. When combined with other anticancer medications, this innovative approach could potentially result in the complete eradication of ovarian cancer. The current studies raise some additional issues that warrant further research. What are the key antigens in ESCs/IPSCs that can trigger effective antitumor immunity across a wide range of cancer types? If these antigens are unique to stem cells, it may raise concerns that vaccination with ESCs/IPSCs and potent vaccine adjuvants will result in some level of autoimmunity against somatic stem cells.

## Figures and Tables

**Table 1:** *In vivo* tumorigenicity of sorted ID8 spheroid cells in NOD/SCID mice.

Number of inoculated cells	Rate of tumor formation				
	CD133^+^	(%)	CD133^−^	(%)	*P*-value (Fisher's exact test, Two-sided)
100	5/5 ^*∗*^	100	0/5	0	0.0079
500	5/5	100	0/5	0	0.0079
2,000	5/5	100	3/5	60	0.4444
10,000	5/5	100	5/5	100	>0.9999

Twenty NOD/SCID female mice were randomly divided into four groups. Various numbers (100, 500, 2,000, and 10,000) of viable CD133-positive and CD133-negative cells in PBS/Matrigel (1 : 1) were subcutaneously injected into the right and left flanks of NOD/SCID mice separately.  ^*∗*^The rate of tumor formation refers to the number of tumors formed/number of inoculated mice.

## Data Availability

Data supporting this research article are available on request.
